# Effect of Extended CT Perfusion Acquisition Time on Ischemic Core and Penumbra Volume Estimation in Patients with Acute Ischemic Stroke due to a Large Vessel Occlusion

**DOI:** 10.1371/journal.pone.0119409

**Published:** 2015-03-19

**Authors:** Jordi Borst, Henk A. Marquering, Ludo F. M. Beenen, Olvert A. Berkhemer, Jan Willem Dankbaar, Alan J. Riordan, Charles B. L. M. Majoie, on behalf of the MR CLEAN investigators

**Affiliations:** 1 Department of Radiology, Academic Medical Center, Amsterdam, the Netherlands; 2 Biomedical Engineering and Physics, Academic Medical Center, Amsterdam, the Netherlands; 3 Department of Radiology, University Medical Center Utrecht, the Netherlands

## Abstract

**Background and Purpose:**

It has been suggested that CT Perfusion acquisition times <60 seconds are too short to capture the complete in and out-wash of contrast in the tissue, resulting in incomplete time attenuation curves. Yet, these short acquisitions times are not uncommon in clinical practice. The purpose of this study was to investigate the occurrence of time attenuation curve truncation in 48 seconds CT Perfusion acquisition and to quantify its effect on ischemic core and penumbra estimation in patients with acute ischemic stroke due to a proximal intracranial arterial occlusion of the anterior circulation.

**Materials and Methods:**

We analyzed CT Perfusion data with 48 seconds and extended acquisition times, assuring full time attenuation curves, of 36 patients. Time attenuation curves were classified as complete or truncated. Ischemic core and penumbra volumes resulting from both data sets were compared by median paired differences and interquartile ranges. Controlled experiments were performed using a digital CT Perfusion phantom to investigate the effect of time attenuation curve truncation on ischemic core and penumbra estimation.

**Results:**

In 48 seconds acquisition data, truncation was observed in 24 (67%) cases for the time attenuation curves in the ischemic core, in 2 cases for the arterial input function and in 5 cases for the venous output function. Analysis of extended data resulted in smaller ischemic cores and larger penumbras with a median difference of 13.2 (IQR: 4.3–26.0)ml (P<0.001) and; 12.4 (IQR: 4.1–25.7)ml (P<0.001), respectively. The phantom data showed increasing ischemic core overestimation with increasing tissue time attenuation curve truncation.

**Conclusions:**

Truncation is common in patients with large vessel occlusion and results in repartitioning of the area of hypoperfusion into larger ischemic core and smaller penumbra estimations. Phantom experiments confirmed that truncation results in overestimation of the ischemic core.

## Introduction

Multiple randomized controlled trials have shown the efficacy of intravenous thrombolysis up to 4.5 hours from onset in patients with acute ischemic stroke [1]. Although convincing evidence is currently lacking, based on experience, patients who do not respond to intravenous thrombolysis or are not eligible for intravenous thrombolysis, may receive intra-arterial treatment (IAT) up to 6 to 8 hours from onset [1]. It has been demonstrated that pre-treatment ischemic core volume is an important predictor of outcome after intra-arterial treatment [2][3]. Although diffusion weighted imaging is the best imaging modality for this purpose [4] its use is currently limited by its unavailability in the acute setting. It has been proposed that CT Perfusion (CTP) parameters like cerebral blood flow (CBF), cerebral blood volume (CBV), mean transit time (MTT) and time to peak (TTP) may potentially be used to estimate areas of irreversible brain damage (ischemic core) and potential salvageable areas of hypoperfusion (ischemic penumbra) [5][6]. Due to its speed, few contraindications for its use [7], and wide availability of CT scanners in emergency departments, CTP has the potential to provide clinical decision support in patients with acute ischemic stroke [8][9]. However, before its acceptance in clinical practice, there are several CTP pitfalls, which compromise accurate CTP analysis that need to be dealt with. Examples of known pitfalls are patient movement [10], errors in placement of arterial input function (AIF) and venous output function (VOF), heterogeneity in thresholds and post-processing [11][12]. A limited acquisition time of <60 seconds is another potential source of error because delayed arrival of contrast agent may result in incomplete capture of the tissue time attenuation curves (TACs) during acquisition [13][14][15]. It is known that truncation of tissue TACs may preclude accurate calculation of CTP parameters [12][16][17]. Despite recommendations of using an acquisition time up to 90 seconds [18], many hospitals still use a potential too short acquisition time of <60 seconds[19][20][21]. Furthermore, the effect of truncation of tissue TACs on ischemic core and penumbra volume estimation is unknown at present.

The aim of this observational case cohort study is to determine the occurrence of tissue TAC truncation in 48 seconds acquisition data. Furthermore, we investigate the effect of truncation using a digital CTP phantom and by comparison of CTP analysis on standard and extended acquisition image data of patients with acute ischemic stroke due to a proximal intracranial arterial occlusion of the anterior circulation.

## Materials and Methods

### Digital head phantom data

**Fig 1:**
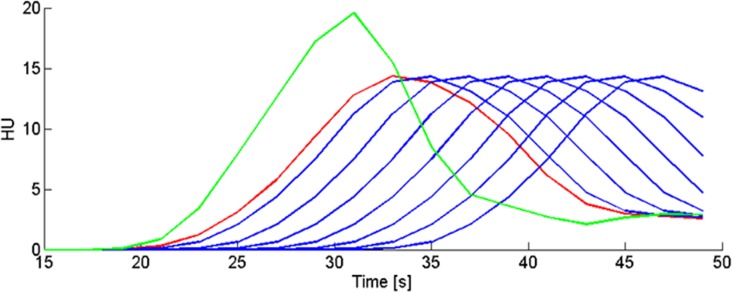
Tissue TACs of the hypoperfused and healthy tissue in the phantom data. In green is the TAC of the healthy tissue. In red is the TAC of the hypoperfused tissue without arrival delay of contrast agent (the original phantom data). The blue curves are the shifted TACs of the hypoperfused tissue of each individual phantom (n = 7). Each curve has a different amount of time shift (from left to right:1, 3, 5, 7, 9, 11 and 13 seconds), and thus arrival delay of contrast agent, relative to the TAC of the healthy tissue (green). With increasing time shift the proportion of the TAC that is truncated increases.

We have used a previously validated digital CTP head phantom [22] to generate a “gold standard” and quantify the effect of tissue TAC truncation on ischemic core and penumbra estimation. Forty-eight seconds CTP data with truncated tissue TACs was created by simulating delayed arrival of contrast agent in the hypoperfused tissue (ischemic core and penumbra). We created the CTP phantom with similar settings as the CTP imaging protocol of hospital A (Table 1). A very small ischemic core (0.1 ml) and large penumbra volume (61 ml) was created at the right hemisphere by applying a mask to the phantom CTP data that designated lower perfusion values for the calculation of the TACs. Eight phantom data sets were constructed, with delay in the arrival of the contrast agent in the hypoperfused tissue ranging from 0 to 13 seconds to simulate different proportions of the TAC being truncated. Arrival delay of contrast agent was simulated by shifting the TACs of the hypoperfused tissue in time relative to the TACs of the healthy tissue (see Fig. 1). The generated image data was suitable for further CTP analysis.

**Table 1:** Scanner and settings.

	Hospital A	Hospital B
Number of patients scanned	28 (patients 1 to 28)	8 (patients 29 to 36)
Scanner	64-slice scanner (sliding gantry Sensation 64, Siemens Medical Solutions, Forchheim, Germany)	128-slice scanner (Philips Brilliance iCT; Best, the Netherlands).
Total acquisition time	210 s	210 s
Standard Acquisition	1 image every 2 s for the first 48 s	1 image every 2 s for the first 48 s
Extended acquisition	60 s after start 1 image every 30 s	60 s after start 1 image every 30 s
Image acquisition parameters	80 kVp, 100 mAs	80 kVp, 100 mAs
Collimation	24 x1.2 mm	128 x 0.625 mm
Brain coverage	28.8 mm	40 mm to 80 mm
FOV	300 mm	220 mm
Reconstructed section width	4.8 mm	5.0 mm
Slice location	At the level of the third ventricle	At the level of the third ventricle
Contrast material	Iopromide (Ultravist 300; Bayer HealthCare Pharmaceuticals, Pine Brook, New Jersey)	Iopromide (Ultravist 300; Bayer HealthCare Pharmaceuticals, Pine Brook, New Jersey)
Contrast volume	40 ml followed by 40 ml of saline	40 ml followed by 40 ml of saline
Injection rate	4 ml/s via 18 G cannula in the right antecubital vein	6 ml/s via 18 G cannula in the right antecubital vein
Start of acquisition	7 seconds after the start of injection of contrast agent	simultaneously with the start of injection of contrast agent

### Patient selection

In our university medical centers, patients with acute ischemic stroke are screened by NCCT. Patients that do not respond to intravenous thrombolysis or are not eligible for intravenous thrombolysis are screened for inclusion in the MRCLEAN trial [23] by CTA and optional CTP. All patients included in the MRCLEAN trial in our university medical centers that underwent CTP with a total acquisition time of 210 seconds were retrospectively included in this study. Patients with a proximal intracranial arterial occlusion of the anterior circulation on CTA are eligible for inclusion in the MRCLEAN. Patients with cerebral ischemia within the previous 6 weeks, and severe head injury in the previous four weeks were excluded.

### Ethics statement

The CTP protocol has been approved by the institutional review board (Medisch Ethische Toetsings Commissie) from the Academic Medical Center, Amsterdam, The Netherlands. Patients or legal representatives signed informed consent.

### CTP imaging protocol

The protocol, as introduced by the Dutch Acute Stroke Trial [24], comprises one image every 2 seconds for the first 48 seconds, followed at 60 seconds after start with a second acquisition of one image every 30 seconds. Resulting in a combined acquisition time of 210 seconds. Details of the protocol can be found in Table 1. The effective dose of the standard CTP acquisition was 0.43 mSv and the total effective dose of the extended acquisition was 0.53 mSv.

### CTP analysis

The CTP analysis was performed by a trained observer (with two years of experience) using Philips software (Philips Extended Brilliance Workspace, version 3.5, Brain CT Perfusion Package, Philips Healthcare, Best, The Netherlands). The default preprocessing steps included filtering, registration and segmentation of brain tissue.

To select the location for the AIF, two regions of interest were selected, containing the anterior cerebral artery or middle cerebral artery (MCA) in the hemisphere contralateral to the occlusion. In each region of interest the voxel with the highest attenuation was automatically selected. Out of these options, the position with the highest attenuation was selected for the AIF [25]. For the VOF, two regions of interest were selected containing either the superior sagittal sinus or straight sinus. Similar to the AIF selection, the position with the highest attenuation was selected for the VOF [26]. The analysis resulted in maps of the CBF, MTT, CBV, and TTP. Calculation of these parameters and determination of the thresholds for ischemic core and penumbra are previously reported [27]. Ischemic penumbra was defined as a relative MTT 50% higher than that of the contralateral hemisphere [28]. Ischemic core was defined as a relative MTT > 1.5 and a CBV lower than 2.0 ml/100g [27].

**Fig 2:**
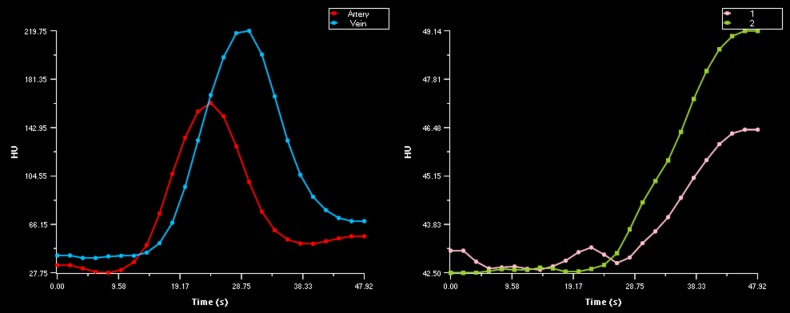
Examples of tissue time attenuation curve truncation with normal AIF and VOF. Normal AIF and VOF for the CTP data of patient 19 (left) and corresponding truncated tissue TAC for the 48 seconds acquisition data of the same patient (right). These figures illustrates that an acquisition time of 48 seconds can be insufficient to capture the complete outwash of contrast agent from the ischemic tissue.

**Fig 3:**
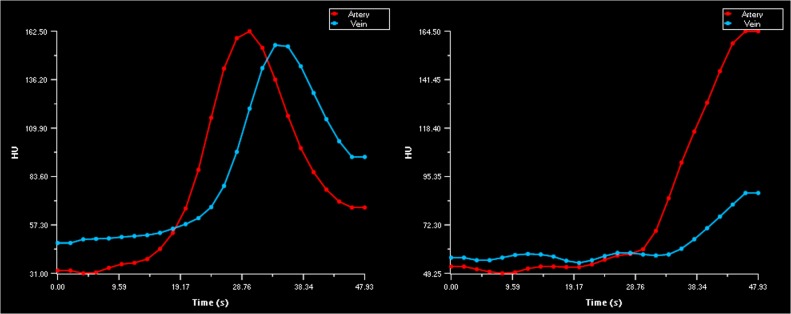
Examples of AIF and VOF truncation. Example of normal AIF curve with truncated VOF curve (left).Example of truncated AIF and VOF curves (right). For both examples longer acquisition time is needed to capture the complete in-wash and outwash of contrast agent.

**Fig 4:**
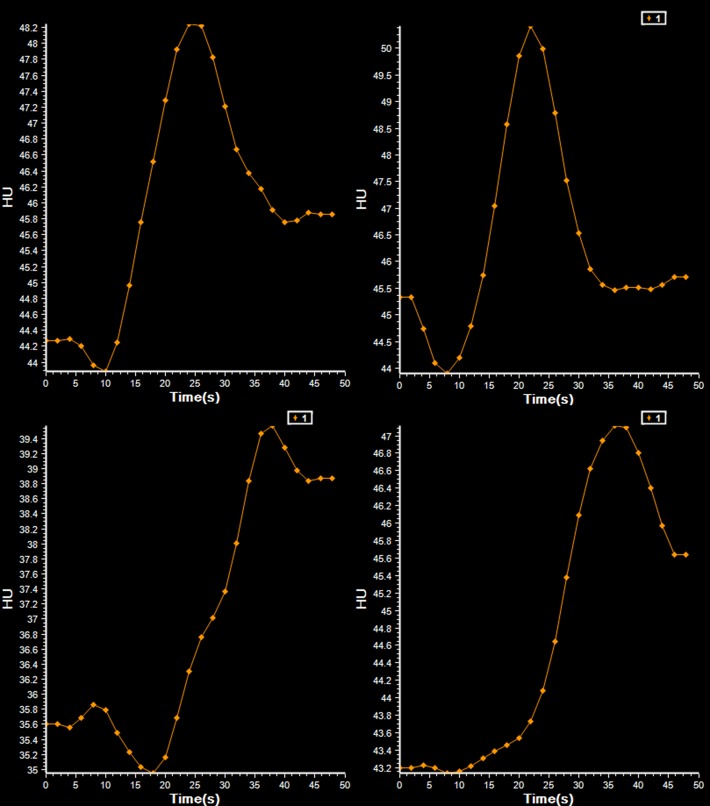
Examples of complete and incomplete tissue time attenuation curves. Complete tissue time attenuation curves from patient 6 (upper left) and patient 24 (upper right). Truncated tissue time attenuation curve from patient 7 (bottom left) and 3 (bottom right). The AIF and VOF of patients 7 and 3 were complete.

CTP analysis was performed on the 48 seconds patient and phantom data. After merging the standard and extended imaging data, the combined 210 seconds patient data was analyzed. To minimize intraobserver variability the AIF and VOF, were chosen in the same vessel and location for the 48 and 210 seconds patient data. Regions of interest were placed in the ischemic core, as defined by the software, to inspect tissue TACs. The TACs for the 48 seconds and 210 seconds image data were classified as complete or truncated in consensus (with 2 years and with more than 10 years of experience). Truncation was defined as an incomplete capture of the TAC either due to incomplete wash-in or wash-out of the first-pass bolus of contrast agent during the acquisition (see Figs. 2–4). Volumes of the ischemic core and penumbra were recorded. For a subset of 18 patients the 48 seconds data was measured a second time. The observer selected the same vessel location for the AIF and VOF as for the first measurement. We also determined whether patients had mismatch, which has been suggested as a selection criteria for therapy [29][30]. Mismatch was defined as an ischemic core < 70 ml and a penumbra of at least 10 ml and 80% larger than the ischemic core [29].

### Statistical analysis

The median and interquartile ranges (IQRs) of paired absolute and relative differences in volume of ischemic core, penumbra, and perfusion abnormality (ischemic core + penumbra) between the data from the standard and extended acquisition time were determined. Median relative difference of the measured volumes was calculated as the paired difference in volume divided by the average volume. The Wilcoxon signed-rank test was used to compare statistical significance of the median differences. P values smaller than 0.05 were considered statistically significant. Intra-observer variability was determined by Bland Altman 95% limits of agreement and calculation of the Intraclass Correlation Coefficient. Statistical analyses were performed using IBM SPSS version 20 (IBM Corp., Armonk, NY, USA).

## Results

### Digital head phantom data

**Fig 5:**
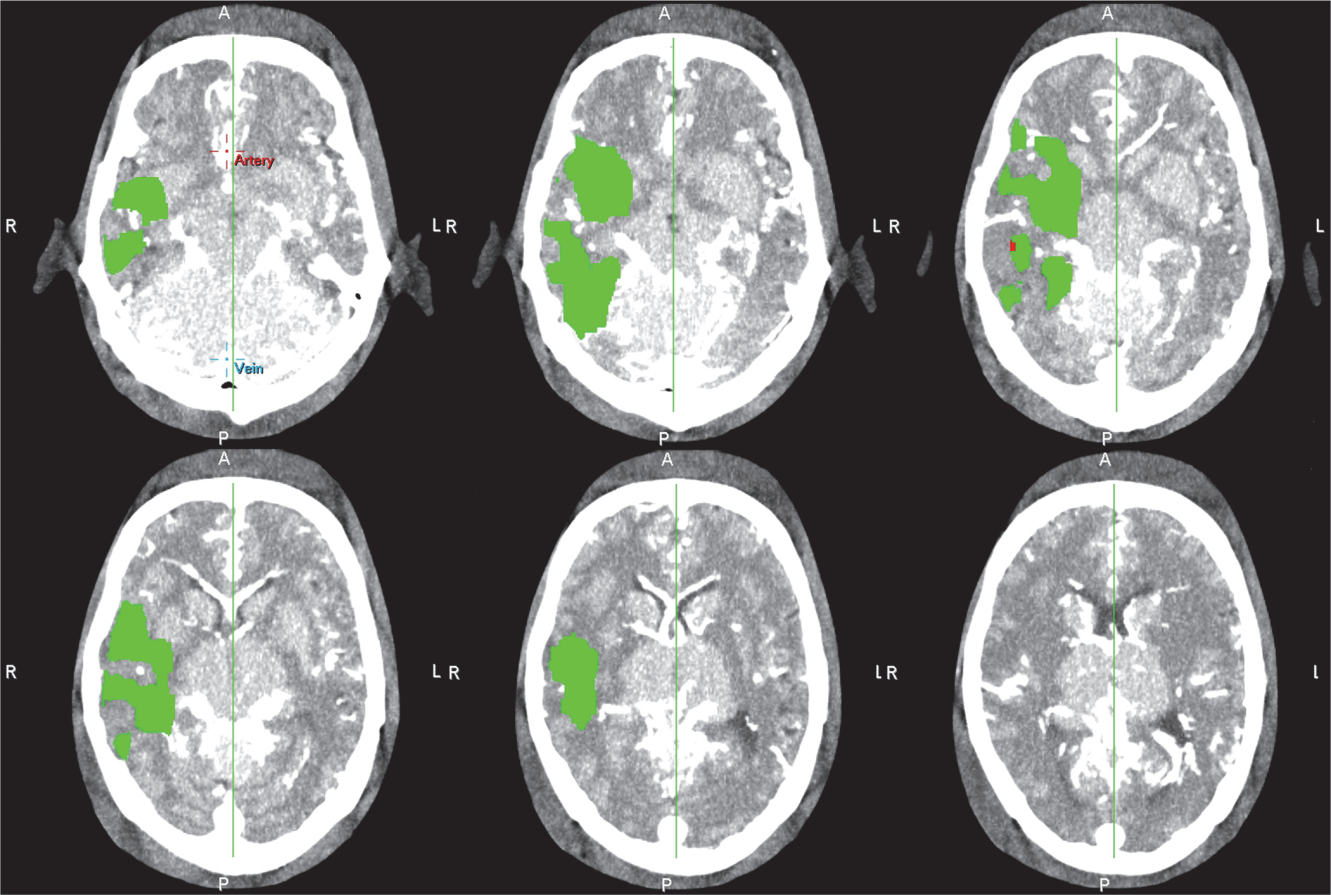
Summary Maps from original phantom data. CTP summary map for all slice locations resulting from analysis of the original CTP phantom data without arrival delay of contrast agent.

**Fig 6:**
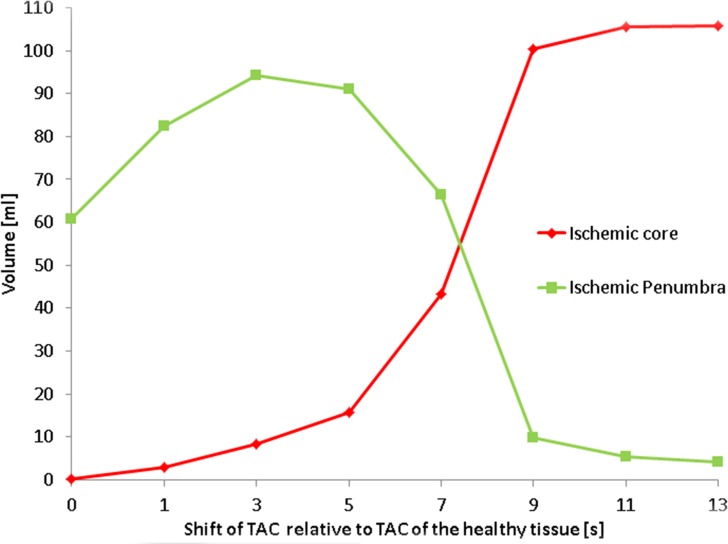
Ischemic core and penumbra from phantom data. This figure shows the effect of shifting TACs of the hypoperfused tissue relative to the TACs of the healthy tissue, and thus simulating contrast arrival delay, on ischemic core and penumbra determination.

A CTP summary map resulting from CTP analysis of the original phantom data, without arrival delay of contrast agent and thus without truncation, is shown in Fig. 5. The volumes of ischemic core and penumbra of the 8 digital phantom data sets, with each a different proportion of the tissue TAC being truncated are shown in Fig. 6. With increasing time shift of the TACs of the hypoperfused tissue the proportion of the TAC that is truncated increases. Fig. 6 shows that for a delay of 3 seconds and more the ischemic core volume increases, and the penumbra volume decreases with increasing delay. Note tissue TAC truncation for a delay of 3 seconds and more in Fig. 1.

### Patient selection

Fifty-eight patients with acute ischemic stroke due to a proximal intracranial arterial occlusion of the anterior circulation underwent CTP analysis with an acquisition time of 210 seconds. Thirteen patients were excluded because of severe head movement during the standard and extended acquisition and five patients were excluded because of movement during the extended acquisition. Four patients were excluded because of impossibility of combining the regular CTP scans with the extended scans in the software due to differences in pixel spacing (N = 2), and insufficient contrast supply (N = 2). The exclusion resulted in 36 patients (mean age: 61 years, age range: 34–86 years, median National Institutes of Health Stroke Scale (NIHSS) 16 (IQR: 12–21)) suitable for analysis. See Table 2 for the patient demographics, clinical characteristics, Alberta Stroke Program Early CT score (ASPECTS), and the measured perfusion defect volumes.

**Table 2:** Patient demographics, clinical characteristics, and the measured perfusion defect volumes.

Patient no.	Sex	Age	NIHSS	Time from onset to imaging [hh:mm]	NCCT ASPECTS	Intracranial Occlusion & side	Truncation	Ischemic core derived from 48 seconds data [ml]	Ischemic core derived from 210 seconds data[ml]	Ischemic penumbra derived from 48 seconds data [ml]	Ichemic penumbra derived from 210 seconds data [ml]	Perfusion Abnormality derived from 48 seconds data [ml]	Perfusion Abnormality derived from 210 seconds data [ml]	Follow up infarct core* [ml]	Atrial fibrillation	Cardiac ejection fraction <40%	Ipsilateral extracranial ICA stenosis or occlusion	Ipsilateral extracranial ICA dissection
1	Male	38	12	01:53	7	M2 R	Tissue TAC	18.2	10.0	59.5	66.0	77.7	76.1	16.3	No	No	No	Yes, (both) sides)
2	Female	66	23	04:39	8	M1 R	Tissue TAC	13.3	3.3	44.4	53.3	57.7	56.5	53.0	Yes	No	No	No
3	Male	61	15	03:00	8	M2 R	Tissue TAC	22.5	8.7	70.3	82.9	92.8	91.7	10.8	No	No	No	Yes, (both sides)
4	Male	67	12	01:17	8	M1 R	VOF	53.2	47.7	3.8	14.5	57.0	62.3	63.5	Yes	No	No	No
5	Male	72	23	00:38	9	M1 L	Tissue TAC	86.7	83.7	3.0	7.4	89.7	91.2	118.8	No	No	No	No
6	Male	54	11	03:15	9	M1 R	No	18.1	9.7	26.7	49.6	44.8	59.2	63.8	No	No	No	No
7	Male	63	6	01:18	10	M1 L	Tissue TAC	25.5	10.0	52.6	68.6	78.1	78.6	Not available	No	No	Yes	No
8	Female	34	18	01:17	8	ICA & M1 L	Tissue TAC	24.6	1.7	46.8	66.9	71.5	68.6	59.9	No	No	No	No
9	Female	67	17	01:54	9	M1 R	Tissue TAC	51.8	43.8	11.8	22.4	63.6	66.2	87.2	No	No	No	No
10	Female	64	13	03:21	10	M2 L	Tissue TAC	0.6	0.0	2 1.5	24.8	22.2	24.8	11.6	No	No	No	No
11	Male	39	21	01:15	5	M1 L	Tissue TAC	25.1	19.6	14.5	13.2	39.6	32.8	35.7	No	No	No	No
12	Female	54	16	04:17	8	M1 L	Tissue TAC	17.6	0.0	61.6	78.3	79.2	78.4	17.3	No	No	No	No
13	Male	52	11	01:43	10	ICA, & M1 L	VOF	33.9	6.8	35.2	64.0	69.1	70.8	20.2	No	No	Yes	No
14	Male	66	22	01:45	10	M L1	VOF	57.1	15.7	19.6	64.3	76.7	80.0	1.4	No	No	No	No
15	Female	86	21	Not available	8	M1 R	AIF &VOF	65.4	34.5	0.9	3.7	66.3	38.2	111.4	Yes	No	No	No
16	Male	82	23	01:16	7	M1 L	AIF & VOF	50.0	12.4	0.0	51.1	50.0	63.4	4.1	Yes	No	Yes	No
17	Female	75	4	Not available	9	M1 R	VOF	12.5	0.6	12.7	24.9	25.2	25.5	8.5	Yes	No	No	No
18	Female	48	12	01:48	10	M1 R	No	0.9	0.2	19.5	23.5	20.4	23.7	1.9	No	No	No	No
19	Male	45	16	01:06	10	M1 R	Tissue TAC	39.0	7.3	40.6	70.1	79.6	77.4	31.5	No	No	No	Yes
20	Female	44	14	03:13	6	M2 L	No	1.6	2.5	69.5	71.4	71.1	73.8	0.0	No	No	No	No
21	Male	49	28	04:31	3	M1 L	Tissue TAC	37.1	19.6	32.4	48.6	69.6	68.1	60.9	No	No	No	No
22	Male	51	25	02:21	8	M1 L	Tissue TAC	17.7	0.1	69.4	86.3	87.1	86.4	45.1	No	Yes	No	No
23	Female	82	20	02:21	10	ICA & M1 R	Tissue TAC	67.8	55.8	16.2	35.0	84.1	90.8	Not available	No	No	No	Yes
24	Female	64	13	01:51	10	m1 L	No	10.4	8.4	36.5	40.1	46.9	48.5	Not available	No	No	Yes	No
25	Male	51	26	02:00	7	m2 L	Tissue TAC	2.2	0.0	44.4	45.2	46.6	45.2	6.0	No	No	No	Yes
26	Female	67	17	02:56	10	a1 L	Tissue TAC	1.0	0.6	0.1	0.3	1.2	0.9	5.4	No	No	No	No
27	Female	47	12	04:10	7	m1 R	Tissue TAC	35.2	13.3	40.6	67.2	75.8	80.5	Not available	No	No	No	No
28	Female	84	19	02:50	8	m1 L	VOF	53.7	0.5	16.6	66.9	70.3	67.4	Not available	No	No	No	No
29	Female	47	13	01:20	9	m1 L	No	11.2	8.4	38.3	39.1	49.4	47.5	9.9	Yes	No	No	No
30	Male	60	16	01:57	4	m1 L	Tissue TAC	93.6	39.4	5.5	67.1	99.0	106.5	227.3	No	Yes	No	No
31	Female	78	12	04:46	6	m1 L	Tissue TAC	41.2	26.2	95.7	110.9	136.9	137.1	192.1	Yes	No	No	No
32	Male	66	16	02:38	10	m2 R	Tissue TAC	70.8	12.5	104.5	156.6	175.3	169.1	40.0	No	No	No	No
33	Female	60	16	02:35	4	m1 L	Tissue TAC	73.3	56.1	78.4	89.6	151.7	145.7	19.0	No	No	No	No
34	Male	58	6	Not available	10	m1 L	Tissue TAC	41.5	12.4	84.6	114.9	126.2	127.2	51.7	No	No	Yes	Yes
35	Female	80	5	02:24	7	M2 L	Tissue TAC	11.2	7.2	136.0	141.7	147.1	149.0	115.3	No	No	No	No
36	Male	70	25	01:14	9	M1 L	Tissue TAC	24.1	11.6	34.2	46.4	58.3	58.0	276.1	No	No	No	No

TAC: tissue attenuation curves, VOF: venous output function, AIF: arterial input function.

* derived from 24 hour or 5/6 days NCCT follow up scan with the same co-registered coverage as the CTP.

### Patient CTP data

In the standard 48 seconds image data, truncation of the VOF curve with truncation of the AIF curve was found in 2 (6%) patients, truncation of the VOF curve without truncation of the AIF was found in 5 (14%) patients. For all patients with AIF and/or VOF truncation, tissue TAC truncation was also observed. Furthermore truncation of tissue TAC, without truncation of the AIF or VOF, was found in 24 (67%) patients. There was no truncation of TACs in the 210 seconds image data.

**Fig 7:**
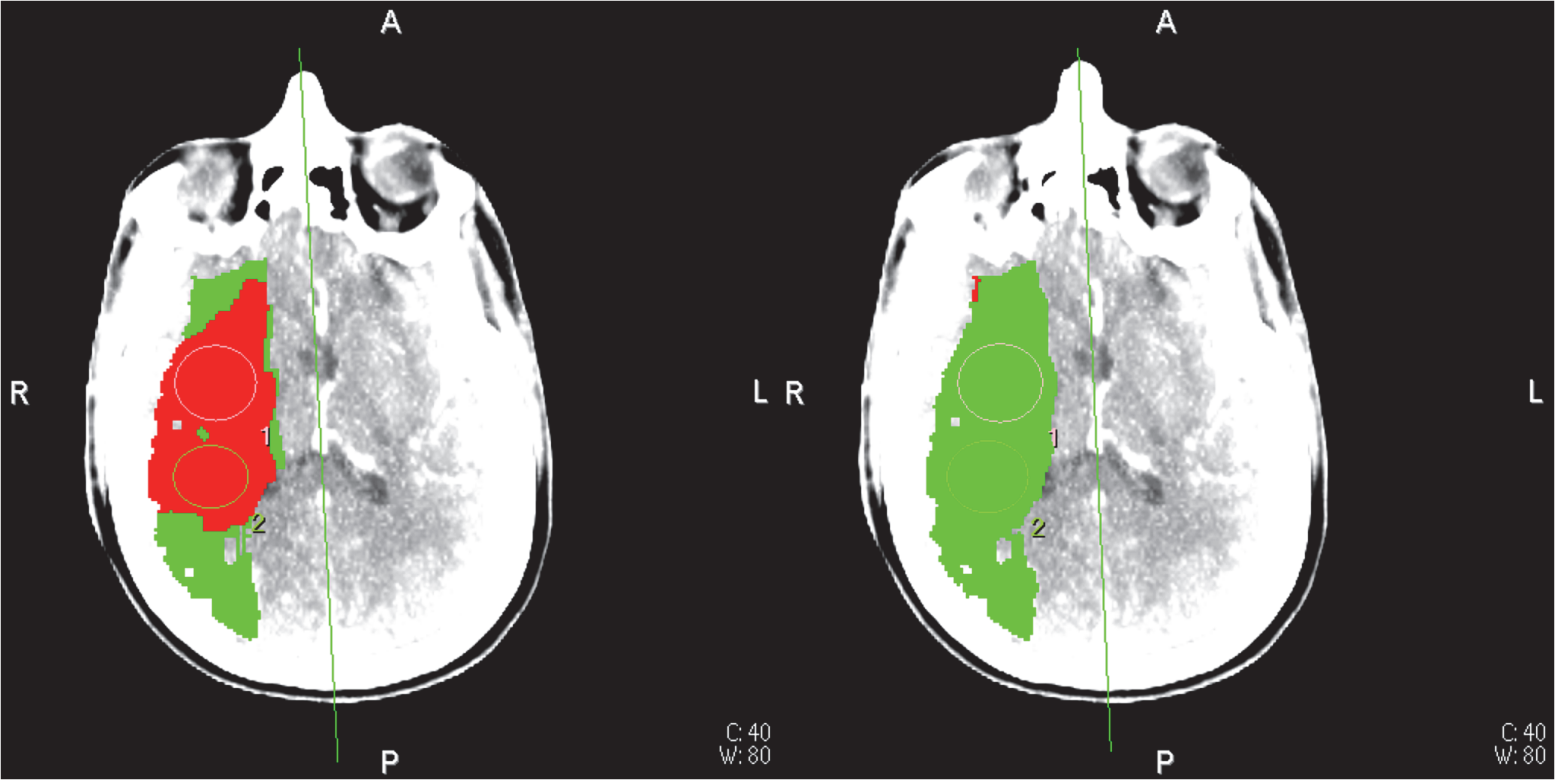
CTP summary maps from patient 19. CTP summary map for the 48 seconds acquisition data with truncated tissue TAC (left). Corresponding CTP summary map for the 210 seconds acquisition data with complete TACs (right). Red: ischemic core, green: ischemic penumbra (See Fig. 2 for corresponding TAC curves).

For 35 out of 36 patients, the ischemic core was smaller for extended CTP acquisition time image data. Fig. 7 shows a typical CTP summary map with its corresponding tissue TACs in Fig. 2. Only for patient 20, the ischemic core was slightly larger with the longer acquisition time.

See Table 3 for the differences in volumes between the extended and standard acquisition time. The ischemic core was smaller, the penumbra larger, and there was no significant difference in total perfusion abnormality for the extended acquisition with a median paired difference of 13.2 (IQR 4.3–26.0) ml (P<0.001), 12.4 (IQR 4.1–25.7) ml (P<0.001), and 0.2 (IQR-1.6–2.7) ml (P = 0.43), respectively. The median relative difference in volume of ischemic core, ischemic penumbra, and total perfusion abnormality was 84.3 (IQR 31.8–136) % (P<0.001), 32.8 (IQR 14.1–69.6) % (P<0.001), 0.7 (IQR-4.0–4.0) % (P = 0.5), respectively.

**Table 3:** Difference in volumes between the extended (210 s) and standard (48 s) acquisition time (extended-standard).

	*Absolute median paired difference*			*Relative median paired difference*		
	*ischemic core (IQR) [ml]*	*ischemic penumbra (IQR) [ml]*	total perfusion abnormality *(IQR) [ml]*	*ischemic core (IQR) [%]*	*ischemic penumbra (IQR) [%]*	total perfusion abnormality *(IQR) [%]*
*All patients (n = 36)*	-13.2 (-26.0 –-4.3)	*12*.*4* (4.1–25.7)	*0*.*24* (-1.6–2.7)	-84.3 (-136 –-31.8)	*32*.*8* (14.1–69.6)	-.*7* (-4.0–4.0)
*Patients without truncation (n = 5)*	-2.0 (-5.6–0.7)	*3*.*6* (1.3–13.4)	-*2*.*7* (-0.2–8.8)	-28.2 (-92.3–9.5)	*9*.*5* (2.3–39.1)	*3*.*7* (-52.0–21.3)
*Patients with only tissue TAC truncation (n = 24)*	-14.4 (-20.9 –-6.1)	*13*.*9* (6.0–19.7)	-*0*.*5* (-1.6–1.8)	-84.3 (-139 –-43.1)	*28*.*4* (14.1–52.3)	-*1*.*2* (-3.9–1.2)
*Patients with VOF or VOF and AIF truncation (n = 7)*	-30.9 (-41.5 –-12.0)	*28*.*8* (10.7–50.3)	*1*.*7* (-2.9–5.3)	-121 (-183 –-61.8)	*117* (65.1–122)	*2*.*5* (-53.0–8.8)
*Hopsital A (28*. *mm coverage) (n = 28)*	-11.9 (-22.6 –-3.6)	*12*.*4* (3.7–22.1)	*0*.*4* (-1.4–3.1)	-89.4 (-164 –-31.2)	*44*.*6* (16.9–72.6)	*1*.*0* (-15.0–4.2)
*Hospital B (40 mm to 80 mm coverage (n = 8)*	-16.1 (-47.9 –-6.0)	*13*.*7* (7.1–46.7)	-*0*.*0* (-5.0–1.7)	-57.3 (-101 –-31.8)	*22*.*5* (6.5–37.5)	*0*.*2* (-3.9–1.2)

* truncation as assessed on the 48 seconds image data.

For patients without any truncation, the ischemic core was smaller for the extended acquisition with a median paired difference of 2.0 (IQR 0.7–5.6) ml (P = 0.14) and a median relative difference of 28.2 (IQR 9.5–92.3) % (P = 0.23). For patients with only truncation of the tissue TAC, the ischemic core was smaller for the extended acquisition with a median paired difference of 14.4 (IQR 6.1–20.9) ml (P<0.001) and a median relative difference of 84.3 (IQR 43.1–139) % (P<0.001). For the data with 28 mm brain coverage (hospital A) the ischemic core was smaller for the extended acquisition with a median paired difference of 11.9 (IQR 3.6–22.6) ml (P<0.001) and a median relative difference of 89.4 (IQR 31.2–164) % (P<0.001). For the data with 40 to 80 mm brain coverage (hospital B) the ischemic core was smaller for the extended acquisition with a median paired difference of 16.1 (IQR 6.0–47.9) ml (P = 0.012) and a median relative difference of, 57.3 (IQR 31.8–101) % (P = 0.012).

The median paired difference between the two repeated measurements was 0.2 (IQR-0.4–2.7) ml for the ischemic core and 0.1 (IQR 0.0–1.5) ml for the penumbra. The Intraclass correlation coefficient was 0.99 for the ischemic core and 0.96 for the penumbra. The Bland Altman 95% limits of agreement between the repeated measurements ranged from −3.8ml–4.9ml for the ischemic core and −3.8ml–4.6ml for the penumbra.

Mismatch was detected for 25 (70%) patients in the extended data series compared to 14 (39%) patients for the standard acquisition time.

## Discussion

This study shows that truncation of TACs is common in patients with acute ischemic stroke due to a proximal intracranial arterial occlusion of the anterior circulation. Tissue TAC truncation caused overestimation of ischemic core and underestimation of ischemic penumbra in phantom experiments. A similar effect is observed in patient data in which extended acquisition time results in complete TACs and repartitioning of the area of hypoperfusion into smaller ischemic core volumes and larger penumbra volumes compared with standard acquisition time.

If mismatch analysis had been used for patient selection, almost one-third of the patients would have been selected based on extended CTP acquisition but rejected for therapy when standard acquisition time was used.

The prevalence of AIF truncation found in our study is similar to the 9% (1/11) reported previously [31]. Prevalence of tissue TAC truncation in our study is much higher than the previously reported of 31% (4/13) in patients with symptoms of MCA occlusion [32], and 9% (1/11) in patients with a proximal occlusion of the anterior circulation [31]. Schaefer et al. [31] used a longer default acquisition time (60 seconds), and a higher injection rate of the contrast agent (7ml/s), which may explain the lower prevalence [33].

CBV is calculated by dividing the area under the tissue TAC by the area under the AIF [27][34]. Truncated TACs have a smaller area under the curve than complete TACs and therefore causes underestimation of CBV and larger ischemic cores. For some patients without truncation, the ischemic core was slightly smaller using longer acquisition times. This may be caused by the software selecting a larger area under the TAC as first pass bolus for the extended acquisition time resulting in larger CBV and smaller ischemic core. However, we believe that the possibility of a larger CBV estimation, found in patients without truncation, due to longer acquisition times cannot explain the large differences in ischemic core size that we have found in patients with truncated TAC. Since the intra-observer variability is small we are convinced that the observer-dependency is minor compared to the differences between standard and extended CTP acquisitions.

The results from this study pertain to the use of Philips software and used parameters to define ischemic core and penumbra. Since CTP analysis methods vary per software vendor [35] we expect that the effect of truncation varies for different software packages and use of different parameters. Truncation of the VOF seems to have little effect on the ischemic core estimation when relative CBF is used as a threshold to define the ischemic core [17].

It would be interesting to investigate what the effect of truncation is when different parameters (e.g. relative CBF or relative CBV) or other software packages are used to define ischemic core and penumbra.

Due to the occlusion causing the ischemic stroke the arrival of contrast agent is delayed and the mean transit time is prolonged in hypoperfused tissue [26], therefore an acquisition time of ∼50 s can be potentially be too short to capture the complete TAC. Delayed arrival of contrast agent in the tissue causes a shift of the TAC towards the end of the acquisition, and may cause TAC truncation. Besides the occlusion causing the stroke arrival delay of contrast agent may have various extracranial causes, for example, low cardiac output, aortic dissection, severe proximal ICA stenosis, and ICA dissection [36][37][38]. Intracranial occlusions can also cause sluggish flow in the ICA and may cause delayed contrast arrival through collaterals [36][37]. Due to an intracranial occlusion, the contrast agent may arrive via the collateral pathway and is delayed compared with regular perfusion [39]. A large occluded proximal intracranial artery may cause more contrast arrival delay compared with a distal occlusion [40]. Therefore, the prevalence of truncation in this study may be higher than in the general stroke population.

This study has a number of limitations. The software uses MTT to define ischemic penumbra, which may result in overestimation of penumbra for patients with an extracranial ICA stenosis [12][41]. The low temporal resolution of the additional acquisition may result in less accurate results [42]. However, because of the dose restrictions it is not ethical to scan such a long time with a high temporal resolution. We used a delay sensitive CTP analysis method, which may contribute to an overestimation of the ischemic core [31]. It is quite possible that the use of delay insensitive method could reduce the difference in ischemic core sizes between the standard and extended acquisition times. An ischemic core measurement “gold standard” like diffusion weighted imaging for comparison with the CTP summary maps was not available. Follow-up non-contrast-enhanced CT scans performed 3–5 days after the CTP were available, but reliable comparison was hampered due to ischemic core growth [43][44]. Therefore we were not able to validate the use of current available thresholds for the extended acquisition with clinical data. The phantom data was used to generate a “gold standard” and enabled us to quantify the effect of tissue TAC truncation on ischemic core and penumbra estimation. Whether the ischemic core volumes of the 48 or 210 seconds data are more accurate, versus a reference standard, will depend on whether there was truncation, and the proportion of the TAC being truncated in the data used to derive the 2.0g/100mL CBV threshold for ischemic core. However truncation causes incorrect CTP analysis and may causes severe ischemic core overestimation. We have used the same thresholds for data from the standard as extended acquisition. These thresholds might not be optimal for data from the extended acquisition and should be validated.

Differences in scan protocols may have resulted in variation in prevalence of truncation, but it is important to note that both scan protocols, with different injection protocols, can result in truncation. For hospital B, which uses a higher injection rate than hospital A, there was no truncation of the AIF or VOF observed. Since the absolute difference in ischemic core between the extended and standard acquisition time was larger for full brain coverage, the small brain coverage of 28.8 mm, may have caused underestimation of the volume differences. A relative small sample size was analyzed and many patients were excluded because of movement. Patients with acute ischemic stroke may be agitated and tend to move which limits the feasibility of CTP [45].

Although strong evidence that patients benefit from IAT after 6 to 8 hours from onset is currently lacking [1], it has been suggested that patients with a large ischemic core (>100 ml) are anyway unlikely to benefit from IAT [46]. Overestimation of ischemic core affects mismatch analysis and may result in incorrectly withholding the patient from treatment. Therefore accurate determination of the ischemic core and penumbra is crucial for CTP to become a standard treatment decision tool in clinical practice. The potential harm of incorrectly withholding a patient from treatment justifies the slightly larger effective dose of the longer acquisition. TAC truncation is common in 48 seconds imaging data and as shown in phantom data results in overestimation of the ischemic core. Longer CTP acquisition time prevents TAC truncation and may improve the accuracy of ischemic core estimation.

## Conclusions

In this study we observed that in 48 second acquisition data, truncation of TACs is common in patients with acute ischemic stroke due to a proximal intracranial arterial occlusion of the anterior circulation. Phantom experiments confirmed that truncation results in overestimation of the ischemic core. By using sufficiently long acquisition times, time attenuation curve truncation can be prevented which results in smaller ischemic core estimations and may affect treatment decisions.
